# Machine Learning–Based Audiovisual Phenotyping for Measuring Communication, Shared Decision-Making, and Trust

**DOI:** 10.2196/85906

**Published:** 2026-03-03

**Authors:** Shely Khaikin, Vineet Tiruvadi, Jeffrey Brooks, Alice Baird, Anne-Catherine Grela-Mpoko, Lindsey Hoffman, Jadyn Crossley, Menachem Leasy, Jaime Fineman, Margot Savoy, Laura Igarabuza, Anuradha Paranjape, Cheryl YS Foo, Michael L Birnbaum, Yaara Zisman-Ilani

**Affiliations:** 1 Shared Decision Making Laboratory, Temple University, Philadelphia, PA, United States; 2 Harvard University, Cambridge, MA, United States; 3 Hume AI, New York, NY, United States; 4 Department of Clinical Family and Community Medicine, Lewis Katz School of Medicine, Temple University, Philadelphia, PA, United States; 5 Department of Clinical Medicine, Lewis Katz School of Medicine, Temple University, Philadelphia, PA, United States; 6 American Academy of Family Physicians, Washington, DC, United States; 7 University of Colorado School of Medicine, Aurora, CO, United States; 8 Center of Excellence for Psychosocial and Systemic Research, Department of Psychiatry, Massachusetts General Hospital, Boston, MA, United States; 9 New York State Psychiatric Institute, New York, NY, United States; 10 Department of Psychiatry, Vagelos College of Physicians and Surgeons, Columbia University, New York, NY, United States; 11 Department of Clinical, Educational and Health Psychology, Division of Psychology and Language Sciences, University College London, 1-19 Torrington Place, London, WC1E 7Hb, United Kingdom; 12 Department of Social and Behavioral Sciences, Barnett College of Public Health, Temple University, Philadelphia, PA, United States; 13 Department of Psychiatry and Behavioral Sciences, Lewis Katz School of Medicine, Temple University, Philadelphia, PA, United States

**Keywords:** audiovisual digital phenotyping, shared decision-making, AI, artificial intelligence, depression, primary care, natural language processing

## Abstract

Machine learning–based audiovisual phenotyping can reveal hidden discrepancies between patients’ self-reported experiences and nonverbal expressions, offering a promising tool for objectively assessing communication quality and advancing health equity.

## Introduction

Although depression is highly prevalent, many patients do not engage with prescribed treatments, particularly racial and ethnic minority individuals in primary care settings where clinicians lack time and infrastructure for effective communication [1]. Shared decision-making (SDM) can enhance engagement [2], but SDM is not yet the norm [3]. Social desirability bias, power dynamics, and cultural norms may lead patients to report high SDM and trust [4] despite feeling otherwise.

Objective measurements may better capture true experiences. Current SDM assessments rely on subjective self-reports or observer ratings with no objective alternatives. Audiovisual digital phenotyping (ADP) is useful in monitoring depression [5,6] and could assess communication quality. This study evaluated multimodal ADP’s usability for assessing health communication, SDM, and trust in depression care. We compared ADP outputs from audio, visual, and language modalities with validated patient-reported measures to identify patterns of discrepancies and alignments.

## Methods

### Study Design

Twenty-four participants were recruited from primary care practices. Eligible adults had a depressive disorder diagnosis (ICD-10-CM F33.xx) and a recent primary care visit. Participants completed recorded video interviews about patient-provider communication, decision-making experiences, and self-report measures: SDM-Q-9-Psy [7] (low to high SDM), CollaboRATE [8] (low to high provider engagement effort), and Trust scale [9] (low to high trust). Mean and sum scores were calculated; participants were categorized as having negative or positive communication experiences based on lower or higher scores, respectively.

From the interviews, two short video clips per participant were extracted: one reflecting positive communication experiences and one reflecting negative experiences. Verbal and nonverbal responses were analyzed with three on-premise Hume AI expression models capturing ADP [10,11]: (1) a facial expression model (FaceNet Inception-ResNet V1) capturing movement and nonverbal expression of the face; (2) a speech prosody model (Whisper-Small), assessing tone and vocal dynamics from audio; and (3) a natural language processing (NLP) model (BERT), identifying the emotionality of the spoken transcript. For each participant, the top three emotions per modality were extracted.

Alignment or discrepancy between self-report and ADP was assessed using face validity by comparing self-report scores with emotional outputs. Participants with positive experiences were matched to positive clips; those with negative experiences were matched to negative clips. Alignment required concordance between reported experience and extracted emotions (eg, negative emotions in negative clips for low scorers). Exploratory analyses examined clips with opposite experience types.

### Ethical Considerations

This study was approved by the Temple University Institutional Review Board (Protocol #29435). Patients provided informed consent for participation. Participants received a US $20 gift card. Self-reported and ADP data were deidentified.

## Results

Of the 24 participants who completed the study, data from 6 were analyzable after excluding cases with simultaneous on-screen appearances of participant and interviewer or poor video quality. The final sample included 3 women (50%), 5 Black participants (83%), and 4 unemployed participants (67%, Table 1). Because the six interviews lasted 48 (SD 13.1) minutes on average, we selected shorter clips for ADP analysis. Selected clip lengths were 14‐58 seconds (mean 29.4, SD 12.7 seconds), each containing approximately 30 analyzable frames per second (about 840‐3480 frames per participant for two clips). Categorization into low and high communication experience was conducted based on an SDM-Q-9-Psy score higher than 2.5 and a Trust score higher than 27.5, as most CollaboRATE scores were above average.

**Table 1.** Demographic characteristics and communication experiences.

Participant characteristics	P1	P2	P3	P4	P5	P6
Age (y)	24	56	58	58	68	39
Sex	Male	Female	Female	Male	Female	Male
Hispanic or Latino	No	No	No	No	Yes	No
Race	Black or African American	White or Caucasian	Black or African American and American Indian/Native American or Alaska Native	Black or African American	Black or African American and Other	Black or African American
Employment status	Employed	Unemployed	Unemployed	Unemployed	Unemployed	Unemployed
Decision made at the consultation	Referral to outpatient center	Refills, no new decisions were made	Referrals	Keep current medication	Stop therapy	Change in medications
SDM-Q-9-Psy, mean score (range 0‐5)	1.11	1.89	3.00	4.56	5.00	5.00
CollaboRATE, mean score (range 0‐9)	5.67	4.00	6.67	9.00	9.00	9.00
Trust in provider, sum score (range 0‐55)	22	24	37	43	54	55

Four participants (P3-P6) reported positive communication experiences. However, for 3 (75%) participants, ADP analysis revealed discrepancies between self-reported positive experiences and the presence of negative (eg, distress or disappointment) or neutral (eg, confusion) emotion outputs in positive clips (Table 2). Disappointment, awkwardness, and annoyance were common negative emotions in negative clips by participants who reported positive overall experiences. These relationship-related emotions may reflect disappointment with specific aspects of the patient-provider communication. Among the ADP modalities, the greatest discrepancies between verbal content and ADP were observed in facial expression and NLP (in positive clips), whereas speech prosody aligned more closely with survey results in 2 participants (P4 and P6), with emotional outputs such as excitement and amusement (Table 2).

**Table 2.** Audiovisual digital phenotyping of emotional outputs.

Participant	Clip type and modality					
	Negative, mean (SD)			Positive, mean (SD)		
	FE^a^	SP^b^	NL^c^	FE	SP	NL
P1^d^	Amusement: 0.41 (0.13) Joy: 0.40 (0.16) Satisfaction: 0.35 (0.11)	Anxiety: 0.18 (0.19) Confusion: 0.16 (0.17) Calmness: 0.15 (0.14)	Confusion: 0.32 (0.22) Anxiety: 0.22 (0.15) Contemplation: 0.18 (0.16)	Amusement: 0.50 (0.20) Joy: 0.50 (0.23) Satisfaction: 0.40 (0.11)	Realization: 0.16 (0.15) Amusement:0.12 (0.10) Disgust: 0.12 (0.12)	Excitement: 0.27 (0.23) Enthusiasm: 0.24 (0.07) Interest: 0.21 (0.13)
P2^d^	Calmness: 0.41 (0.17) Tiredness: 0.36 (0.16) Boredom: 0.32 (0.08)	Awkwardness: 0.20 (0.14) Sadness: 0.17 (0.19) Realization: 0.14 (0.11)	Annoyance: 0.31 (0.18) Disappointment: 0.24 (0.14) Pain: 0.16 (0.18)	Confusion: 0.35 (0.13) Concentration: 0.33 (0.15) Calmness: 0.31 (0.15)	Disappointment: 0.29 (0.30) Confusion: 0.25 (0.14) Realization: 0.21 (0.13)	Disapproval: 0.31 (0.25) Disgust: 0.24 (0.34) Annoyance: 0.20 (0.13)
P3^e^	Confusion: 0.49 (0.14) Doubt: 0.33 (0.09) Distress: 0.28 (0.08)	Realization: 0.27 (0.17) Distress: 0.19 (0.15) Awkwardness: 0.17 (0.05)	Annoyance: 0.36 (0.18) Disappointment: 0.32 (0.17) Sadness: 0.22 (0.19)	Confusion: 0.43 (0.11) Concentration: 0.34 (0.12) Calmness: 0.32 (0.13)	Distress: 0.21 (0.28) Disappointment: 0.19 (0.16) Realization: 0.16 (0.09)	Annoyance: 0.16 (0.06) Anxiety: 0.14 (0.13) Disappointment: 0.13 (0.12)
P4^e^	Confusion: 0.40 (0.15) Disappointment: 0.33 (0.05) Sadness: 0.33 (0.08)	Awkwardness: 0.18 (0.11) Realization: 0.17 (0.11) Calmness: 0.12 (0.16)	Awkwardness: 0.50 (0.10) Anxiety: 0.23 (0.17) Annoyance: 0.19 (0.11)	Pain: 0.56 (0.19) Sadness: 0.50 (0.10) Distress: 0.46 (0.09)	Determination: 0.19 (0.19) Excitement: 0.16 (0.25) Amusement: 0.15 (0.19)	Awkwardness: 0.31 (0.07) Realization: 0.20 (0.03) Doubt: 0.18 (0.12)
P5^e^	Confusion: 0.35 (0.11) Distress: 0.29 (0.08) Pain: 0.27 (0.18)	Realization: 0.12 (0.13) Amusement: 0.12 (0.14) Sadness: 0.11 (0.22)	Sadness: 0.43 (0.31) Disappointment: 0.23 (0.14) Annoyance: 0.20 (0.21)	Confusion: 0.39 (0.11) Distress: 0.30 (0.06) Disappointment: 0.30 (0.08)	Realization: 0.14 (0.06) Contemplation: 0.12 (0.09) Awkwardness: 0.12 (0.07)	Disappointment: 0.16 (0.16) Realization: 0.16 (0.06) Contemplation: 0.14 (0.04)
P6^e^	Interest: 0.42 (0.05) Amusement: 0.39 (0.13) Concentration: 0.33 (0.09)	Anger: 0.19 (0.23) Contemplation: 0.17 (0.11) Disgust: 0.13 (0.12)	Contemplation: 0.18 (0.14) Emphatic pain: 0.15 (0.18) Sympathy: 0.12 (0.13)	Confusion: 0.48 (0.04) Concentration: 0.44 (0.05) Doubt: 0.37 (0.04)	Realization: 0.21 (0.11) Amusement: 0.13 (0.07) Positive surprise: 0.13 (0.13)	Gratitude: 0.43 (0.28) Relief: 0.23 (0.14) Satisfaction: 0.20 (0.10)

a FE: facial expression (FaceNet Inception-ResNet V1).

b SP: speech prosody (Whisper-Small).

c NL: natural language (BERT).

d Low SDM-Q-9-Psy score.

e High SDM-Q-9-Psy score.

Two participants (P1 and P2) reported negative communication experiences on surveys. In their negative clips, NLP and prosody reflected these experience (eg, anxiety), while facial expressions showed mixed patterns: P1 displayed positive emotions (eg, amusement) and P2 displayed neutral emotions (eg, calmness). In positive clips, P1 showed predominantly positive emotions across all modalities, whereas P2 displayed a mix of neutral and negative emotions (eg, confusion) across all modalities, indicating a discrepancy with the positive clip classification but alignment with P2’s overall negative self-reported communication experience. Notably, P1 exhibited similar facial expressions across positive and negative clips.

## Discussion

This pilot study demonstrated the usability of multimodal ADP for evaluating patient-provider communication, SDM, trust, and engagement, with prosody showing the strongest alignment with self-reported experiences and facial expression showing the weakest alignment. Discrepancies between self-reports and nonverbal expressions may help explain high rates of service disengagement and treatment nonadherence among patients, whose nonverbal communication cues may be clinically overlooked despite reported trust and engagement [12]. Nonverbal expressions aligned with self-reports for negative experiences but contradicted self-reports for positive experiences, highlighting the need for providers to be mindful of social desirability bias and patient-provider power imbalances.

To protect privacy, analyses used on-premises technology, which offers fewer advantages than cloud-based artificial intelligence (AI) models. This created challenges with simultaneous on-screen appearances, poor lighting, and nonstandard camera angles, resulting in a reduced sample size for comparing ADP with SDM and trust measures. Despite constraints, ADP provided hundreds of thousands of analyzable frames per clip, offering extensive repeated measurements. Postappointment data collection was another limitation.

Technologically, facial expression sensitivity in depression requires optimization, as limited facial expression may affect provider responses and ADP emotion extraction. Future research should address how to implement commercial AI tools while respecting ethical requirements when handling protected health information [13]. Additional considerations for on-premises AI studies should ensure sufficient computing capacity to support analyses.

Given our predominantly Black patient sample, findings highlight providers’ need to recognize how social desirability bias, power dynamics, and cultural norms may lead patients to report positive experiences despite feeling disengaged. This demonstrates multimodal ADP’s promise for objectively assessing communication quality and advancing health equity.

## Abbreviations

ADP: audiovisual digital phenotyping
AI: artificial intelligence
NLP: natural language processing
SDM: shared decision-making
